# Review of the anticancer properties of 6‐shogaol: Mechanisms of action in cancer cells and future research opportunities

**DOI:** 10.1002/fsn3.4129

**Published:** 2024-03-27

**Authors:** Gabriela Figueroa‐González, Laura Itzel Quintas‐Granados, Octavio Daniel Reyes‐Hernández, Isaac H. Caballero‐Florán, Sheila I. Peña‐Corona, Hernán Cortés, Gerardo Leyva‐Gómez, Solomon Habtemariam, Javad Sharifi‐Rad

**Affiliations:** ^1^ Laboratorio de Farmacogenética, UMIEZ, Facultad de Estudios Superiores Zaragoza Universidad Nacional Autónoma de México Ciudad de México Mexico; ^2^ Colegio de Ciencias y Humanidades, Plantel Cuautepec Universidad Autónoma de la Ciudad de México Ciudad de México Mexico; ^3^ Laboratorio de Biología Molecular del Cáncer, UMIEZ, Facultad de Estudios Superiores Zaragoza Universidad Nacional Autónoma de México Ciudad de México Mexico; ^4^ Departamento de Farmacia, Facultad de Química Universidad Nacional Autónoma de México Ciudad de México Mexico; ^5^ Laboratorio de Medicina Genómica, Departamento de Genómica Instituto Nacional de Rehabilitación Luis Guillermo Ibarra Ibarra Ciudad de Mexico Mexico; ^6^ Pharmacognosy Research & Herbal Analysis Services UK Central Avenue, Chatham‐Maritime Kent ME4 4TB UK; ^7^ Facultad de Medicina Universidad del Azuay Cuenca Ecuador

**Keywords:** 6‐Shogaol, cancer, drug resistance, ginger

## Abstract

Cancer is a major global health challenge that affects every nation and accounts for a large portion of the worldwide disease burden. Furthermore, cancer cases will rise significantly in the next few decades. The Food and Drug Administration has approved more than 600 drugs for treating diverse types of cancer. However, many conventional anticancer medications cause side effects, and drug resistance develops as the treatment proceeds with a concomitant impact on patients' quality of life. Thus, exploring natural products with antitumor properties and nontoxic action mechanisms is essential. Ginger (*Zingiber officinale Roscoe*) rhizome has a long history of use in traditional medicine, and it contains biologically active compounds, gingerols and shogaols. The main ginger shogaol is 6‐shogaol, whose concentration dramatically increases during the processing of ginger, primarily due to the heat‐induced conversion of 6‐gingerol. Some studies have demonstrated that 6‐shogaol possesses biological and pharmacological properties, such as antioxidant, anti‐inflammatory, and anticancer activities. The mechanism of action of 6‐shogaol as an anticancer drug includes induction of paraptosis, induction of apoptosis, increase in the production of reactive oxygen species, induction of autophagy, and the inhibition of AKT/mTOR signaling. Despite this knowledge, the mechanism of action of 6‐shogaol is not fully understood, and the scientific data on its therapeutic dose, safety, and toxicity are not entirely described. This review article examines the potential of 6‐shogaol as an anticancer drug, addressing the limitations of current medications; it covers 6‐shogaol's attributes, mechanism of action in cancer cells, and opportunities for future research.

## Graphical abstract

This review article considers the following facts: (i) the current necessity for the development of natural anticancer medication, (ii) the ability of cancerous cells to develop resistance against conventional drugs, and (iii) that 6‐shogaol has been shown to have effects against the progress of cancer in studies in vivo (in animal models). The comprehensive review aims to synthesize and analyze the progress in the knowledge of 6‐shogaol as a potential anticancer drug and identify opportunities to continue the research.

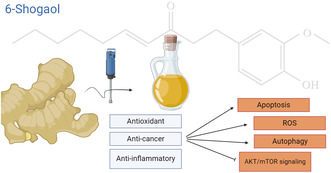

## 1 INTRODUCTION

Cancer remains one of the world's most serious public health problems, both in disease burden and contributor to death (Kocarnik et al., 2022; Siegel et al., 2022; Sung et al., 2021). A significant increase in cancer cases is also expected in the coming decades due to an aging population, accelerated socio‐economic development, population growth, smoking, sedentary lifestyles, and obesity, among others. Thus, it is necessary to continue searching, developing, and characterizing new drugs with anticancer properties to face this growing health problem.

Developing new drugs is time‐consuming and expensive, often taking more than 10 years from initial discovery to marketing. In addition, success rates in oncology therapeutics are low due to toxicities observed during preclinical and clinical trials (Kumari & Dang, 2021). Currently, the Food and Drug Administration (FDA) has authorized around 650 anticancer drugs for treating specific types of cancer, such as pembrolizumab for esophageal cancer, tamoxifen citrate for breast cancer, nelarabine for lymphoblastic leukemia, and bevacizumab for liver cancer, among others (National Cancer Institute, 2023).

However, many conventional anticancer drugs produce side effects and suffer from drug resistance (Basak et al., 2021). Cancer drug resistance can occur for many reasons, including problems with drug intake, compartmentalization, and metabolism. Drug resistance mechanisms can also involve activating alternate pathways, changes in membrane lipids and target proteins, apoptosis blockade, and increased drug removal from cells (Dallavalle et al., 2020; Holohan et al., 2013). In this context, it is essential to explore natural products with antitumor properties and nontoxic action mechanisms (Holohan et al., 2013; Nedungadi et al., 2018).

Ginger (*Zingiber officinale Roscoe*) rhizome has an ancient use in traditional medicine (Nedungadi et al., 2018). Its phytochemical components are diverse and include phenolics and terpenes such as zingiberene, curcumins, gingerols (6‐gingerol, 8‐gingerol, and 10‐gingerol), and shogaols (6‐shogaol) (da Silveira Vasconcelos et al., 2019). Some studies have shown that 6‐shogaol produces biological and pharmacological properties such as antioxidant, antilipidemic, antihyperglycemic, anti‐inflammatory, antimicrobial, and anticancer activities (Ali et al., 2008; Ballester et al., 2022).

The mechanism of action of 6‐shogaol as an anticancer drug includes induction of paraptosis (a nonapoptotic path effective in cancer cell death) in breast cancer cells (Nedungadi et al., 2018), induction of apoptosis in liver cancer cells via the inactivation of Wnt (Zhang et al., 2021), the increase in the production of reactive oxygen species (ROS) in human colorectal carcinoma cells (Pan et al., 2008), induction of autophagy in MCF‐7 cancer stem cells, and the modulation of notch or the inhibition of AKT/mTOR signaling in lung cancer A549 cells (Hung et al., 2009; Ray et al., 2015). 6‐Shogaol lacks a formal establishment of the mechanism of action; thus, it is necessary to focus on this topic.

This review article considers the following facts: (i) the current necessity for the development of natural anticancer medication, (ii) the capacity of cancerous cells to develop resistance against conventional drugs, and (iii) that 6‐shogaol has been shown to have effects against the progress of cancer in studies in vivo (in animal models). The comprehensive review aims to synthesize and analyze the progress in the knowledge of 6‐shogaol as a potential anticancer drug and identify opportunities to continue the research.

## 2 REVIEW METHODOLOGY

To condense the advances and progress in the knowledge of 6‐shogaol as a future anticancer drug, we considered and reviewed specialized information in electronic web databases: Google Scholar, PubMed, Embase, Scopus, and Medline. The search was performed to cover a period until June 2023. The MeSH for searching using “AND” and “OR” was intended to know the sources, phytochemistry, and relationship between the chemical structure of 6‐shogaol and the anticancer activity. We used MeSH terms “rhizome,” “ginger,” “*Zingiber officinale Roscoe*,” “chemical structure,” “6‐shogaol,” “sources,” “traditional medicine,” “ethnobotany,” and “anticancer activity.” Furthermore, we searched the semi‐synthetic derivatives using “6‐shogaol derivatives” and “6‐shogaol synthetic”. In pharmacology, we were interested in the bioavailability and pharmacokinetics of 6‐shogaol. Consequently, we used the following words in the search “concentrations,” “rats,” “animals,” “humans,” “dose,” “bioavailability,” and “pharmacokinetics.” Moreover, we recorded the studies as follows: characteristics of the population studied (model animals or humans), the dose used (mg/kg/day), via and time (day) of administration, the pharmacokinetic parameters such as peak concentration (C_max_; ng/mL), time of peak concentration (T_max_; h), and elimination half‐life (*t*_1/2_). We converted the units reported in some articles in the C_max_, T_max_, and *t*_1/2_ to homogenize all studies' presentations when necessary.

To design our research question and accomplish our literature review for preclinical antitumor studies, we employed the PICO acronym [P as the population, I as the intervention, C as the comparators, and finally, O as the outcomes] framework from evidence‐based medicine (Speckman & Friedly, 2019). Our investigation question was as follows: What are the doses and the mechanism of action of 6‐shogaol that produce an antitumor effect under in vitro and in vivo studies? We included only original articles and considered animal models or studies that used cell cultures as the population. We considered the administration in animals or exposure in cells to 6‐shogaol as an intervention. As the comparator, we considered only articles that presented a control, placebo, or sham group. Finally, as the outcome or results, we considered the main effect in the progression or inhibition of cancer; we included the IC_50_/dose reported by the authors, the action mechanism, and the signaling pathways related to 6‐shogaol to produce their antitumor effect. To learn about the human clinical studies associated with 6‐shogaol as an antitumor compound, we searched at https://www.clinicaltrials.gov/. We registered the current state of clinical trials, population characteristics, and results.

To acquire data about the antitumor mechanism of action of 6‐shogaol, toxicity, safety, and secondary/side effects, we examined the articles used in the sections described above. We searched in existing reviews toward the use of the MeSH “toxicity,” “6‐shogaol,” “secondary OR side effects,” “safety,” “harmless,” “toxicology,” “action mechanism,” “antitumor,” and “*Zingiber officinale Roscoe*” to complement the information. Finally, we included 79 articles for information inserted in the body text and discussion.

We included peer‐reviewed articles in English; we excluded theses and reports of Congress. In the case of the review of preclinical antitumor studies, we excluded: (i) noncontrolled studies, (ii) studies made with other components of ginger as gingerols or another shogaol, and (iii) articles that do not show complete information. All articles were preliminarily reviewed by title and summary to choose the material for the present review.

## 3 SOURCES, PHYTOCHEMISTRY, AND CHEMICAL STRUCTURE–ANTICANCER ACTIVITY RELATIONSHIP

The phenolic alkanone 6‐shogaol belongs to the family of compounds identified as shogaols, the dehydrated form of gingerols (Kou et al., 2018). Ginger is the source of gingerols and 6‐shogaol; however, 6‐shogaol cannot be found in fresh ginger in high amounts and must be produced by low‐temperature drying (Kou et al., 2018). Shogaols extraction can be performed from oils of dried ginger or directly from ginger previously dried through convective drying processes such as freeze drying, room‐temperature drying, solar exposure, microwave drying, and intermittent microwave (Kou et al., 2018). It is documented that 6‐shogaol can increase from 0.09 mg/g in fresh ginger to 0.209–0.384 mg/g after drying (Kou et al., 2018). The extraction procedure is completed by reflux, shaking at room and warm temperatures, sonication, high‐pressure Soxhlet extraction, or supercritical fluid extraction (Žitek et al., 2022). Inside the extract, the shogaols with higher activity are in less quantity than gingerols. The extraction conditions (e.g., drying temperatures and pH) can be optimized to obtain extracts rich in 6‐shogaol (Huang et al., 2011; Ok & Jeong, 2012). Another way to increase the amount of 6‐shogaol is by converting 6‐gingerol through steam heating. The manipulation of ginger at 120°C for 4 h leads to a conversion of 36.78% of 6‐gingerol to 6‐shogaol, with a loss of 6‐, 8‐, and 10‐gingerols over 50% after 2 h under steaming (Cheng et al., 2011; Zhang et al., 2006). Ok and Jeong (2012) optimized the procedure for extracting 6‐shogaol as the significant component of the ginger extract by slicing the rhizome, letting it dry in a convection oven at 80°C, grounding that into a fine powder, and extracting the different compounds with ethanol at a pH of 1 and 80°C in reflux for 24 h; this ethanol was further partitioned with water/DCM and the organic phase was dried out by evaporation. An HPLC of the different extraction conditions tested confirmed the results. These high yields could be achievable since 6‐gingerol suffers dehydration under acidic conditions, which converts it to 6‐shogaol (Kou et al., 2018). This process can also be done under alkaline conditions, but the degradation rate decreases significantly (Ok & Jeong, 2012).

Additionally, Permatasari et al. (2022) purified an extract with a 94% yield of 6‐shogaol by macerating rhizomes with methanol; this solvent is suitable for the extraction of phenolic and flavonoid compounds (Salih et al., 2021) and sonicating it for 3 h, this methanolic extract was then processed under a vacuum liquid chromatography method using a dual eluent of hexane and ethyl acetate, the fraction with a more significant amount of 6‐shogaol was the one with polarity hexane:ethyl acetate 9:1. This extract was then passed through an HPLC semi‐prep, where the highest purification percentage was achieved—moreover, Rigane et al. (2018) succeeded in purifying 6‐shogaol from a methanolic extract of ginger using a silica gel column, which was eluted with a mixture of hexane and diethyl ether (7:3). Unfortunately, this procedure had a yield of 1.5%. However, these are better results than the ones reported by Mallavadhani et al., where a methanolic extract of the rhizome was passed through a silica gel column. Nevertheless, they used a gradient elution of hexane and ethyl acetate, yielding 0.25% (Rigane et al., 2018).

In recent years, 6‐shogaol has been characterized as a chemical compound with promising biological activity against various cancers, including leukemia, liver, lung, colon, breast, gastric, skin, kidney, ovarian, and prostate. The cellular processes associated with the molecular anticancer action of 6‐shogaol include the activation of cell death through apoptosis, autophagy, necrosis, and mitotic catastrophe. Furthermore, 6‐shogaol promotes the anticancer mechanism through tumorigenesis and tumor progression suppression (Kou et al., 2018). In this regard, the chemical structure of 6‐shogaol plays a crucial role in the effectiveness of their mechanism of action in the different types of cancer.

**FIGURE 1:**
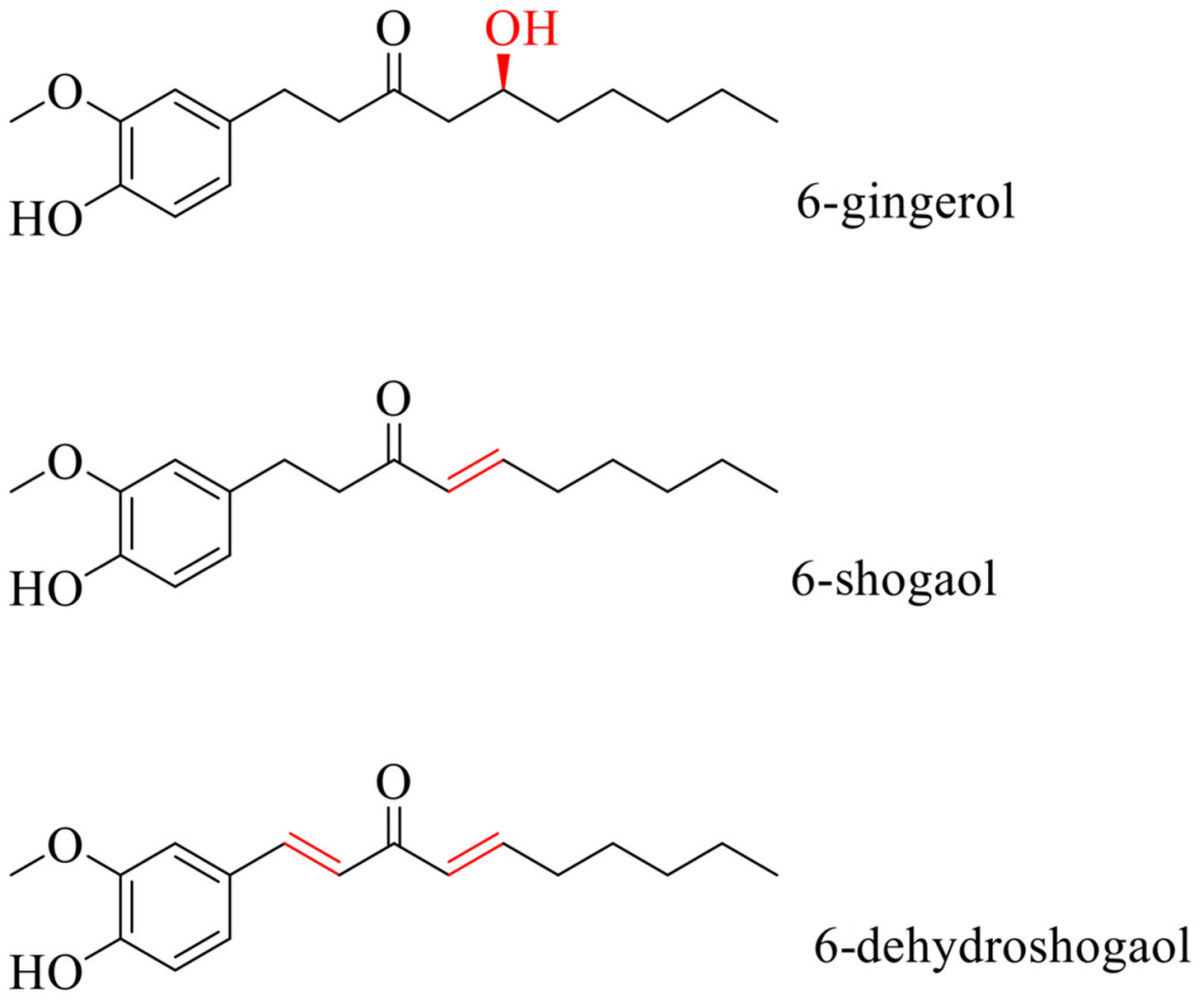
Molecular structure of 6‐gingerol, 6‐shogaol, and 6‐dehydroshogaol. Dehydration reaction such as treatment by heat converts 6‐gingerol to 6‐shogaol. The α,β‐unsaturated structural moiety in 6‐shogaol makes it more biologically active as an anticancer agent than 6‐gingerol.

The 6‐shogaol or 1‐(4‐hydroxy‐3‐methoxyphenyl)‐4‐decen‐3‐one (Figure 1) presents a molecular weight of 276.4 g/mol. The structure has a – C = C – adjacent to the carbonyl group, and this double bound distinguishes the 6‐shogaol from the 6‐gingerol (Ishiguro et al., 2008). The presence of this α,β‐unsaturated carbonyl structural moiety corresponds to the 3‐nonen‐2‐one and gives the 6‐shogaol the ability to inhibit tubulin polymerization (IC_50_ = 32 μM in HGC 27 cells), while 6‐gingerol did not. Thus, 6‐shogaol causes cell cycle arrest during the M phase, interfering with microtubule dynamics and resulting in mitotic catastrophe (Ishiguro et al., 2008). The inhibition of tubulin polymerization is found in the reaction between the sulfhydryl groups of the cysteine residues in tubulin with the α, β‐unsaturated carbonyl in the 6‐shogaol (Ishiguro et al., 2008). However, this reaction depends on the chain length of the α,β‐unsaturated carbonyl in the 6‐shogaol (Ishiguro et al., 2008). The reaction between 6‐shogaol and the cysteine residues is a Michael addition.

The Michael acceptors, such as the 6‐shogaol, are soft electrophiles, easy to modify cysteine residues covalently (soft nucleophiles) in signaling proteins such as reduced glutathione (GSH) (Zhang et al., 2013). Furthermore, this kind of interaction is a class of pro‐oxidants because the covalent modification of GSH or thioredoxin reductase (TrxR) leads to an imbalance of cellular redox homeostasis (Yan et al., 2016). In this regard, Peng et al. (2023) explored the inhibition of TrxR by 6‐shogaol on three enzyme preparations: NADPH‐reduced recombinant rat TrxR1 (WT TrxR1); 2) U498C TrxR1 in which Sec498 was exchanged with Cys residue; and 3) glutathione reductase (Peng et al., 2023). 6‐Shogaol specifically inhibited the activity of WT TrxR. Likewise, the researchers tested the possible interaction of mammalian TrxR with 6‐shogaol by docking. The study highlighted that the α,β‐unsaturated carbonyl in the 6‐shogaol covalently bonded to TrxR1 at Sec498 (Peng et al., 2023). The binding place of the 6‐shogaol was described in two parts, namely Trp407, Leu409, Glu494, Cys497, and Sec498 on chain A and Ala26, Lys29, Tyr116, Ile347, and Arg351 on chain B (Peng et al., 2023). The chemical interactions involved in the binding process include conventional hydrogen bonds with Trp407, Gly499, Sec498, and Tyr116, carbon–hydrogen bonds with Leu409, Glu494, and Cys497, and alkyl bonds with Ala26, Lys29, Ile347, and Arg351 (Peng et al., 2023).

On the other hand, 6‐shogaol may inhibit the Akt kinase activity, a downstream mediator of the epidermal growth factor receptor (EGFR). Akt kinase is constitutively active in non‐small‐cell lung cancer cells (NSCLC) and is a downstream effector of PI3K. The overactivation of Akt is a common molecular characteristic associated with several cancers (Song et al., 2019). In this respect, Kim et al. (2014) elucidated the mechanism of 6‐shogaol linked to inhibition of the Akt activity. The authors identified the exact sites of binding where 6‐shogaol binds to Akt1, which is located underneath the activation loop Akt1 (Kim et al., 2014). The allosteric binding site of the Akt1 is placed at the lower interface between the N‐ and C‐lobes of the kinase domain (Kim et al., 2014). 6‐Shogaol can form two hydrogen bonds with Ser205, and the presence of this residue is crucial to these bonds. In addition, there is a strong hydrophobic interaction between 6‐shogaol and Leu210, Ile290, Leu275, Leu261, Tyr272, and Leu264 residues from the kinase domain of Akt1, as well as hydrophobic interactions between the phenyl ring of 6‐shogaol and Trp80 in the PH domain of Akt1 (Kim et al., 2014). Also, Mizra et al. recently described a predominant hydrophobic interaction between 6‐shogaol and Ala177 at 3.9 Å by protein–ligan interaction profiler (Mirza & Karim, 2023). Collectively, all these drug–target interactions enhance the inhibition of 6‐shogaol against Akt.

With another approach, Mulati et al. (2022) elucidated the ability of the 6‐shogaol to interact with heat shock proteins (HSPs) in NSCLC (Mulati et al., 2022). The HSPs are essential in cellular homeostasis and are upregulated in malignant tumor cells (Mulati et al., 2022). The variant HSP60 is directly related to the occurrence, development, and metastasis in tumors, and it is highly expressed in tumoral cells, including breast cancer, NSCLC, and ovarian cancer (Mulati et al., 2022). The authors estimated the potential position of 6‐shogaol binding on HSP60 through the Amber 18 program (Mulati et al., 2022). This strategy allowed them to describe the top 10 amino acids (ILE 50, PRO 33, MET 482, SER 151, GLY 53, GLY 154, ASN 153, GLY32, THR 90, and THR 159) that contribute to the binding of 6‐shogaol and HSP60 (Mulati et al., 2022). A hydrophobic interaction among 6‐shogaol, ILE 105, and PRO33 was also critical in binding (Mulati et al., 2022). However, the hydrogen bonds formed with GLY53 of HSP 60, parallel with hydrophobic interactions with THR 90, ILE 150, and PRO 33, indicated the main binding forces (Mulati et al., 2022).

6‐Shogaol is also involved in the inflammasome innate immune pathway. Inflammasomes are cytoplasmic multimeric protein complexes that are paramount in activating the innate immune system, allowing optimal signaling and avoiding deviant activation (Martinon, 2019). It efficiently regulates processes like inflammation, production of ROS (Zhao et al., 2020), and secretion and rifting of proinflammatory cytokines by the caspase‐1 pathway, as well as virus infection management (Wofford et al., 2020). In this regard, Kode et al. (2023) assessed the antiviral and anti‐inflammatory properties of 6‐shogaol during a SARS‐CoV2 infection and its role in the NLRP3 inflammasome route in various cell lines. For Vero cells, 6‐shogaol demonstrated a successful inhibition of the viral infection in a dose‐dependent manner, with an EC50 of 0.95 μg/mL; meanwhile, the positive control compound, remdesivir, exhibited an EC50 of 0.90 μg/mL (Pruijssers et al., 2020). It also was demonstrated that 6‐shogaol manages to inhibit the production of ROS from the mitochondria and the expression of the AIM2/NLRP3 and caspase‐1 pathway in the A594 cell line with a concentration of 5 μM.

These results are of great concern because it has been seen that during severe COVID‐19 cases, a cytokine storm (Zhao et al., 2021) is produced by a greater activation of the inflammasome pathway. This causes programmed cell death and the recruitment of various immune cells, which also liberates more cytokines into the bloodstream, producing a proinflammatory feedback loop. Overall, the overactivation of this inflammation route causes severe damage to the lung structure (Kode et al., 2023), decrementing the patients' quality of life after recovery. By administering this phytochemical, it is expected that the management of the infection will be more controllable, reducing many of its side effects.

## 4 COMPUTATIONAL BINDING (MOLECULAR DOCKING)

Molecular docking is an in silico technique that allows studying the interaction between a molecule of interest and its receptor so that the complex is in a state of minimum energy, hence the most probable conformation (Meng et al., 2011). This tool is vital in studying new drugs or their repositioning (Luo et al., 2016).

Kode et al. (2023) established that 6‐shogaol interacts with the NLRP3 inflammasome, specifically in the ADP‐binding cavity, thus inhibiting the protein complex activity related to the proinflammatory cascades (Zhao et al., 2021). It was also shown that 6‐shogaol interacts with the cavity predominantly by hydrophobic bonds and with H‐bonds to a lesser extent. The principal amino acids that interacted with the compound were A227 and A228.

Furthermore, Gratal et al. (2022) demonstrated that 6‐shogaol bonded to the hydrophobic pocket on the protein myeloid differentiation factor 2 (MD‐2) in the toll‐like receptor (TLR)4/MD‐2 receptor and inhibited its signaling cascade since this pocket is much broader than the molecule, they achieved to have docked up to three molecules inside that space. This receptor is a crucial component in the innate immunity response, ultimately releasing proinflammatory molecules and tissue destruction related to osteoarthritis. This disease is caused by wrong receptor activation due to mechanical, physiological, and biological changes in the joint, which can increase with age (Barreto et al., 2020).

## 5 SEMI‐SYNTHETIC DERIVATIVES

The synthesis of 6‐shogaol derivatives as dehydroshogaols can be obtained by the Aldol condensation reaction of vanillin acetone with different alkyl aldehydes (Lu et al., 2014). The reactions by typical Michael acceptor as the aryl‐1,4‐dien‐3‐ones through Horner–Emmons olefination of 2‐oxo‐3‐alkenylphosphonates or by conjugate addition of vinyl iodide to styryl‐activated enones under n‐butyllithium (n‐BuLi) require complex and expensive conditions of the reaction. The 6‐shogaol derivatives are relevant due to their increased activity compared to their precursors, the 6‐gingerol and 6‐shogaol. An example of this is the increased pro‐oxidative capacity or the 6‐dehydroshogaol. Liu et al. (2022) investigated the mechanism of action for reducing angiogenesis through the comparative analysis of 6‐gingerol, 6‐shogaol, and 6‐dehydroshogaol (Liu et al., 2022). The study found a higher capacity of 6‐dehydroshogaol to inhibit the cellular migration and invasion of people's umbilical vein endothelial cells. Furthermore, the authors correlated the effect of Michaelis acceptor units in 6‐dehydroshogaol over the depletion of GSH and the inhibition of TrxR (Liu et al., 2022).

One of the drawbacks of 6‐shogaol is its instability at room temperature, which increases the necessity to obtain new derivatives. One target of the 6‐shogaol derivatives is activating the nuclear erythroid two p45‐related factor 2 (Nrf2). The activation of this protein triggers the action of the antioxidant response element (ARE) sequence and leads to the transcription of cytoprotective and detoxifying genes (Sussan et al., 2009; Zhang, 2006); this makes Nrf2 an attractive target for preventing cancer development. 6‐Shogaol derivatives may react with the cysteine residues of the Kelch‐like ECH‐associated protein 1 (Keap1), which disrupts the complex Keap1‐Nrf2, to activate Nrf2 (Zhu et al., 2016). 6‐Shogaol analogs denominated shogaol thiophene compounds (SCTs), obtained by replacing the pentyl group in the said chain with thiophene derivates, are also related to the activation of Nrf2 (Mak et al., 2023). The SCT activity was explored in murine hepatoma cells (Hepa1c1c‐7) by the induction activity and expression of Nrf2 by NAD(P)H quinone oxidoreductase 1 (NOQ1) (Mak et al., 2023). The results indicated that SCT activity could be observed from the submicromolar range while 6‐shogaol was in micromolar concentration. Furthermore, SCTs present higher metabolic stability (Mak et al., 2023).

## 6 BIOAVAILABILITY AND PHARMACOKINETICS OF 6‐SHOGAOL

Several recent studies have been conducted on 6‐shogaol due to its many phytochemicals and potential pharmacological benefits. Unfortunately, few studies are available on its bioavailability and pharmacokinetic parameters (Table 1) (Bao et al., 2020; Zick et al., 2008).

**TABLE 1:** Summary of pharmacokinetic parameters in studies in vivo with 6‐shogaol.

Characteristics of the population studied	6‐Shogaol dose (mg/kg/d); administration^a^	AUC	T_max_ (h)	C_max_ (ng/mL)	*t*_1/2z_ (h)	Reference
Male Sprague–Dawley rats; 220 ± 20 g	Micelles loaded with 6‐shogaol; 5 mg/mL, in normal saline, 100 mg/kg; p.o.	AUC_0–4 h_ (h/ng/mL) 6834.10 ± 826.10	0.92 ± 0.14	1284.3 ± 191	4.65 ± 0.39	Zhang et al. (2019)
	Free 6‐shogaol; 5 mg/mL, in 0.5% (v/v) castor oil mixture, 100 mg/kg; p.o.	AUC_0–4 h_ (h/ng/mL) 2123.20 ± 193.60	0.33 ± 0.14	1085.3 ± 230.1	2.05 ± 0.09	
Wistar rats	Ginger oleoresin; 300 mg/kg in olive oil; p.o.	AUC_0‐t_ (μg/min/mL) 97.80 ± 9.89	0.30 ± 0.00	1502 ± 111	0.43 ± 0.02	Nikam et al. (2013)
Male Sprague–Dawley rats; 236–315 g	^14^C‐6‐shogaol; 3, 10, or 30 mg/kg in propylene glycol/ethanol/water = 2:1:1; p.o.	AUC_inf_ (μg/h/mL): 3.27 ± 0.74; 18.98 ± 6.42; 56.24 ± 4.69 (3, 10, and 30 mg/kg, respectively)	0.25 ± 0.00; 0.42 ± 0.14; 0.67 ± 0.29 (3, 10, and 30 mg/kg, respectively)	1.63 ± 0.08 μg eq/mL; 6.09 ± 1.71 μg eq/mL; 14.50 ± 2.76 (3, 10, and 30 mg/kg, respectively)	4.35 ± 0.01; 4.73 ± 1.23; 3.99 ± 0.16 (3, 10, and 30 mg/kg, respectively)	Asami et al. (2010)
	6‐Shogaol; 40 mg/kg in 2% Tween 80/physiological saline; p.o.	AUC_inf_ (ng h/mL): 148.46 ± 38.36	0.25 ± 0.00	99.99 ± 39.13	2.32 ± 1.79	
Sprague–Dawley rats; 220 ± 20 g	6‐Shogaol; 200 mg/kg in sodium carboxymethylcellulose (0.5%, CMC‐Na); p.o.	AUC_0‐t_ (μg/min/mL): 382.80 ± 47.24	0.5	2230 ± 160	3.25 ± 0.91	Bao et al. (2020)
	6‐Shogaol liposome; 200 mg/kg; p.o.	AUC_0‐t_ (μg/min/mL): 1077.79 ± 45.86	1	4040 ± 150	4.82 ± 0.46	
	TPGS‐coated 6‐shogaol liposomes; 200 mg/kg; p.o.	AUC_0‐t_ (μg/min/mL): 2220.41 ± 24.21	1	5090 ± 240	6.08 ± 0.47	
Human healthy volunteers	Powder ginger capsules; 2 g; p.o.	AUC (μg/mL): 10.9 ± 13.0	1.09 ± 0.37	150 ± 120	2.0 ± 0.7	Zick et al. (2008)

*Note*: p.o., oral; TPGS, aqueous‐soluble derivative of vitamin E, D‐α‐tocopheryl polyethylene 16 glycol (PEG) succinate carrier.

^a^ Administered as one dose.

The effectiveness of 6‐shogaol as an anticancer treatment is hindered by its low solubility in water and rapid metabolism, making it difficult to administer orally and limiting its bioavailability and clinical applications. Indeed, the oral administration of 300 mg/kg of ginger oleoresin comprising 2.03% w/w of 6‐shogaol (around 6 mg/kg) reaches <0.05% of the administered dose in plasma, liver, small intestine, and stomach. These are organs where the 6‐shogaol was mainly distributed, thus confirming its low bioavailability (Nikam et al., 2013); furthermore, according to a study by (Zhang et al., 2019), free 6‐shogaol is quickly broken down in the liver and eliminated through the kidney. The study found a significant accumulation of free 6‐shogaol in the stomach, small intestine, and liver within the first hour due to the liver's first‐pass effect. However, the distribution of free 6‐shogaol in the liver decreased significantly after 2 h (Zhang et al., 2019). Asami et al. (2010) investigated the excretion and metabolic routes of 6‐shogaol using: 6‐shogaol ^14^C labeled and without the label. The authors observed that around 90% of the dose administered orally (3, 10, or 30 mg/kg) of 6‐shogaol ^14^C labeled was taken in through the digestive tract and mainly eliminated through excretion in the bile. Similar results were obtained in the nonlabeled 6‐shogaol; thus, the authors concluded that 6‐shogaol is primarily metabolized in the body and excreted as metabolites (Asami et al., 2010).

Another study evaluated the plasma concentration (ng/mL) after intravenous 6‐shogaol injection (1 mg/kg). At 0.08 h, 6‐shogaol reached a C_max_ of around (50 ng/mL). Moreover, this study observed a collaborative interaction between ginger biophenolics (Gundala et al., 2014); in this respect, it has been reported additive and/or synergistic interactions among the active ginger constituents (6‐gingerol, 8‐gingerol, and 6‐shogaol) to inhibit prostate cancer cell proliferation in an in vitro study (Brahmbhatt et al., 2013). Other ginger biophenolic compounds show different parameters to 6‐shogaol; for example, Ding et al. (1991) observed that during the administration of a bolus intravenous dose of 3 mg/kg, 6‐gingerol was eliminated from the plasma with a total body clearance of 16.8 mL/min/kg and a *t*_1/2_ 7.23 min. Moreover, 6‐gingerol was bound to serum protein by 92.4% (Ding et al., 1991).

On the other hand, encapsulation in nanosystems of 6‐shogaol to improve oral bioavailability has been reported in the last decade. Zhang et al. (2019) used micelles loaded with 6‐shogaol (Ms6), prepared by the nanoprecipitation method. The authors observed an increase of 3.2‐fold in the oral bioavailability of Ms6 compared with the free 6‐shogaol in male Sprague–Dawley rats. In addition, Ms6 presented a significant increase in the in vitro cytotoxic activity compared with the free compound. It also had a hepatoprotective effect in Kunming mice and could significantly improve the tissue distribution of 6‐shogaol in the liver and the brain. According to the authors, micelles can enhance the effectiveness of 6‐shogaol in treating cancer and protecting the liver. It also improves the solubility and oral absorption of 6‐shogaol and its distribution in the brain (Zhang et al., 2019).

Also, 6‐shogaol‐loaded liposomes with an aqueous‐soluble derivative of vitamin‐E, D‐α‐tocopheryl polyethylene glycol (PEG) succinate (TPGS) as a carrier, have been tested (Bao et al., 2020). The results indicate that oral administration of liposomes loaded with 6‐shogaol coated with TPGS or not had relative oral bioavailability of 580.04% and 281.55%, respectively (Bao et al., 2020). Moreover, there was an improvement in the *t*_1/2_, C_max_, AUC_0‐t_, and T_max_ as compared with the free 6‐shogaol. Thus, TPGS‐coated 6‐shogaol liposomes might act as a promising vehicle for brain delivery in the future and could potentially improve the oral bioavailability of lipophilic drugs in vivo (Bao et al., 2020). In addition to using the nanoparticles for 6‐shogaol administration, ginger has been administrated in capsules to evaluate the pharmacokinetics of 6‐shogaol. In humans, ginger was orally administered in single doses of 100 to 2000 mg, and blood samples were obtained in the first 3 days after the administration (from 15 min to 72 h). During the study, no participant had detectable levels of free 6‐shogaol. However, the 6‐gingerol sulfate conjugate was detected at doses >1 g (Zick et al., 2008). Moreover, in most people administered, shogaol conjugates were cleared from the plasma after 4 h (Zick et al., 2008). In another study in humans, after 1 h of oral administration of 2.0 g of ginger extracts that contained 45.04 mg of 6‐shogaol, it was detected (i) free (13.6 ± 6.9 ng/mL), (ii) glucuronidated (0.73 ± 0.54 μg/mL), and (iii) sulfated 6‐shogaol (0.047 ± 0.035 μg/mL) (Yu et al., 2007).

The metabolic fate of the main components of 6‐shogaol has yet to be fully understood. In a study conducted by Chen et al. (2012), the metabolism of 6‐shogaol was analyzed in mice and cancer cells, administering 200 mg/kg of 6‐shogaol in corn oil via p.o. Thirteen metabolites were identified, over half obtained from fecal samples from mice treated with 6‐shogaol and four in cancer cells. The authors indicated that 1‐(4‐hydroxy‐3‐methoxyphenyl)‐decan‐3‐ol (M9) and 1‐(4‐hydroxy‐3‐methoxyphenyl)‐decan‐3‐one (M11) are the bioactive compounds inhibiting cancer cell growth and inducing apoptosis in human cancer cells (Chen et al., 2012).

In summary, a primary challenge in using 6‐shogaol as an antitumor drug is improving its bioavailability for oral administration. Moreover, given the lack of detectable free 6‐shogaol in a human study (Zick et al., 2008), and considering that only the free drugs have a physiologic effect, it could be necessary to perform multidose studies to evaluate the pharmacokinetics parameters. Likewise, considering the additive and/or synergistic effect between ginger compounds observed in the above‐mentioned in vitro studies (Brahmbhatt et al., 2013), it would be convenient to conduct more studies that evaluate the 6‐shogaol with other components of ginger as antitumor compounds. Finally, conducting additional studies on bioavailability and pharmacokinetics can enhance our understanding of the mechanism of action and therapeutic benefits of 6‐shogaol.

## 7 PRECLINICAL ANTICANCER STUDIES

According to GLOBOCAN, 19.3 million new cancer cases were registered worldwide in 2020. Breast cancer is the most commonly diagnosed type of cancer (11.7%), followed by lung (11.4%), colorectal (10.0%), prostate (7.3%), and stomach (5.6%) cancers (Sung et al., 2021).

Recent in vitro studies have converged on the potential function of 6‐shogaol in treating breast, lung, prostate, colon, and liver cancer, among others. Interestingly, some of the investigations address the antitumor properties of this phytochemical from cell lines to animal models to increase understanding of the complex mechanism of 6‐shogaol in cancer cells (Table 2).

**TABLE 2:** Preclinical antitumorigenic studies of 6‐shogaol.

Type of cancer	Model in vitro using cell lines in vivo using animal models	Concentrations IC_50_/doses	Mechanisms/signaling pathways	Results	Ref.
Cervical cancer	Hela and SiHa cell lines	IC_50_ = 25.68 ± 0.47 μM (for HeLa cells) IC_50_ = 37.52 ± 1.56 μM (for SiLa cells)	PI3K/Akt/mTOR pathway	Upregulates the expression of p21, Bax, PARP, and cytochrome c and downregulates the expression of cyclin B1, CDC25A, and Bcl‐2. It induces apoptosis independent of autophagy through the ROS‐mediated PI3K/Akt/mTOR pathway	Pei et al. (2021a)
	Female BALB/c 18 nude mice subcutaneously injected with HeLa cells (1 × 10^7^ cells/mL)	12.5 and 50 mg/kg of body weight	Inhibition of tumor growth and induction of apoptosis	Induces tumor growth inhibition and apoptosis. No changes were found in the serum of 6‐shogaol mice	Pei et al. (2021a)
Ovarian cancer	A2780, OVCAR‐3, Caov‐3, and SK‐OV‐3 cell lines	10, 20, 30, 40, and 50 μM	Induction of ER stress and cell death through upregulation of Nox4 and ROS releasing	Induces the expression of GRP78, p‐PERK, p‐eIF2α, ATF‐4, CHOP, and DR5. In gefitinib‐resistant ovarian cancer cells, 6‐shogaol upregulates N‐cadherin, vimentin, Slug, and Snail, while downregulated E‐cadherin overcoming gefitinib resistance	Kim and Lee (2023)
	BALB/c nude mice (nu/nu) model generated by A2780 cells (1 × 10^7^) injection	40 mg/kg and 60 mg/kg of body weight	Prevention of tumor growth	Reduces tumor volume by sixfold at the dose of 60 mg/kg	Kim and Lee (2023)
	A2780 cell line	IC_50_ = 30 μg/mL for 24 h of incubation IC_50_ = 25 μg/mL for 48 h of incubation	Modulation of the JAK/STAT3 signaling pathway	Induces apoptosis and cytotoxicity, increases ROS production, and inhibits STAT3 translocation, inhibiting the expression of PCNA, cyclin‐D1, and Bcl‐2 but diminishing the expression of Bax, caspase‐3, and caspase‐9	Liang et al. (2019)
Breast cancer	MCF‐7	IC_50_ = 7.94 ± 0.57 μM (monolayer cells) IC_50_ = 39.52 ± 0.62 μM (spheroid)	Modulation of the Notch pathway	Interferes with the Notch pathway causing apoptotic cell death	Ray et al. (2015)
	MDA‐MB‐231	5 to 30 μM	Regulation of the NF‐κB signaling	Inhibits PMA‐induced cell invasion	Ling et al. (2010)
	MDA‐MB‐231	0.1–10 μM	Inhibition of the secretion of CCL2 by TADCs	Inhibits the phosphorylation of STAT3 and decreases the CCL2 expression	Hsu et al. (2015)
	BALB/c mice	30 mg/kg of body weight	Inhibition of transcription factor STAT3 pathway activation in TADCs	Prevents tumorigenesis and metastasis	Hsu et al. (2015)
Endometrial cancer	Ishikawa cell line	IC_50_ = 24.91 μM	Inhibition of cell growth, cell cycle arrest at the G2/M phase, and induction of apoptosis via ER and mitochondrial pathways	Enhances ROS production and activated ER response biomarkers associated with mitochondria‐mediated apoptosis	Ma et al. (2020)
	Female BALB/c nude mice injected with Ishikawa cells (1 × 10^7^/0.1 mL	50 mg/kg of body weight	Inhibition of tumor growth and induction of apoptosis via ER and mitochondrial pathways	Enhances ROS production and triggered apoptosis through ER and mitochondrial pathways	(Ma et al., 2020)
Lung cancer cells	A549	0.1–10 μM	Inhibition of the secretion of CCL2 by TADCs	Inhibits the phosphorylation of STAT3 and decreases the CCL2 expression	Hsu et al. (2015)
	C57BL mice	30 mg/kg of body weight	Inhibition of transcription factor STAT3 pathway activation in TADCs	Prevents tumorigenesis and metastasis	Hsu et al. (2015)
Non‐small‐cell lung cancer	NCI‐H1650 and NCI‐H520	20 μM	PI3‐K/Akt signaling pathway	Inhibits NSCLC growth and arrests cell cycle (G1 phase for NCI‐H1650 cells and G2/M phase for NCI‐H520 cells	Kim et al. (2014)
	Female BALB/c (nu/nu) mice	Implantation of NCI‐H1650 cells (3 × 10^6^ cells) by subcutaneous injection	PI3‐K/Akt signaling pathway	Reduces the tumor xenograft growth of NCI‐H1650 by decreasing cell proliferation and increasing apoptosis	Kim et al. (2014)
	A549 cells	IC_50_ = 55.4 μM	Inhibition of AKT/mTOR pathway	Induces autophagic cell death	Hung et al. (2009)
	A549 cells	IC_50_ = 48.67 μM for 24 h IC_50_ = 77.33 μM for 48 h IC_50_ = 111.33 μM for 72 h	Induction of apoptosis and arrest of the cell cycle at the G0/G1 phase through disrupting the mitochondrial function	Binds to HSP60 resulting in a inhibition of ERK, Stat3, PI3K, Akt, and mTOR signaling pathways	Mulati et al. (2022)
	BALB/c‐nude mice injected with A549 cells (1 × 10^7^)	Combined treatment with 6‐shogaol (5, 10, or 20 mg/kg/day) and taxol (5 mg/kg/day) for 2 weeks	Inhibition of tumor growth	Decreases the tumor volume, and the combinatory treatment with taxol enhanced the antitumor activity of 6‐shogaol	Mulati et al. (2022)
Human colorectal cancer	HCT‐116 and SW‐480	IC_50_ = 7.5 μM for HCT‐116 cells IC_50_ = 10 μM for SW‐480 cells	Inhibition of cell proliferation. Arrest cell cycle through p53/p21 pathway Induce apoptosis via the mitochondrial pathway	Increases p53, p21^waf1/cip1^, and GADD45α and decreases cdc2 and dc25A	Qi et al. (2015)
	Female xenograft mice model (using 2 × 10^6^ HCT‐116‐Luc cells)	Treated consecutively for 5 weeks with 15 mg/kg administrated intraperitoneally each 24 h	Upregulation of the p53‐dependent pathway	Induces apoptosis and arrests cell cycle at G2/M phase and modulates the expression of p21^waf1/cip1^, cdc2, and cdc25A	Qi et al. (2015)
	SW480 and SW620 cell lines	IC_50_ = 20 μM	Induction of cell death associated with activation of autophagy‐ and apoptosis‐related pathways	In single or in combinatory treatments Inhibits cell growth and induces cell death	Woźniak et al. (2020)
Liver cancer	Huh7 Hep3B HepG2 cell lines	5, 10, and 20 μM	Induction of apoptosis via p53 and ROS generation	Induces the expression of p53, LC3‐II, and p62 and, the breakdown of caspase‐3 and ‐9	Nazim and Park (2019)
	HepG2 cells	5, 10, 20, 50, 75, 100, and 200 μM of 6‐shogaol micelles and free form	6‐Shogaol‐loaded micelles improved the inhibition of cell growth compared with free 6‐shogaol	Inhibits the growth of cancer cells more effectively when loaded in micelles than in its free form due to the endocytosis of micelles. Besides, micelles cytotoxicity was higher than free form	Zhang et al. (2019)
Prostate cancer	LNCaP, DU145, and PC3	10, 20, and 40 μM	Regulation of the NF‐κB signaling	Inhibits JAK2 and Src kinases, regulating the phosphorylation of STAT3	Saha et al. (2014)
	HMVP2 (prostate cancer cell line from mice)	10, 20, and 40 μM	Induction of apoptosis	Reduces the phosphorylation of STAT3 and reduces the NF‐κB activation (constitutive and TNF‐α induced)	Saha et al. (2014)
	Syngeneic FVB/N male mice injected with HMVP2 cells	50 and 100 mg/kg body weight	Induction of apoptosis in HMVP2 cells	Reduces the phosphorylation of STAT3 leading to a downregulation of pSTAT3, cyclin D1, and survivin	Saha et al. (2014)
Head and neck cancer	Hep‐2 (squamous cell carcinoma of the larynx cell line)	IC_50_ = 20 μM	Inhibits cell proliferation through ROS‐dependent mitochondrial‐mediated apoptosis	Causes a loss of the mitochondrial membrane potential, DNA, nuclear fragmentation, and oxidative DNA damage	Annamalai et al. (2016)
	SCC25 and CAL27 (squamous cell and carcinoma cell lines of the tongue)	IC_50_ = 6.6 ± 1.06 μM (SCC25) IC_50_ = 8.9 ± 1.09 μM (CAL27)	Induction of apoptosis and enhancement of radiation sensitivity	Decreases the amount of survivin	Kotowski et al. (2018)

Abbreviations: Akt1, protein kinase B or PKB 1; Akt2, protein kinase B or PKB 2; ERK, extracellular signal‐regulated kinases; Hes1, hairy and enhancer of split; HSP60, heat shock protein; IKK, inhibitor of nuclear factor kappa‐B kinase; IL, interleukin; IκBα, inhibitor of nuclear factor kappa‐B alpha; JNK, c‐Jun N‐terminal kinase; LC3, microtubule‐associated protein light chain 3; MMP‐2, metalloproteinase‐2; MMP‐9, metalloproteinase‐9; NF‐κB, nuclear factor kappa‐B; Notch, Notch signaling pathway; PI3K, phosphoinositide 3‐kinase; PUMA, p53 upregulated modulator of apoptosis; STAT3, signal transducer and activator of transcription 3; TNF‐α, tumor necrosis factor‐alpha; XIAP, X‐linked inhibitor of apoptosis protein.

The following sections analyze the properties of 6‐shogaol treatments in cell lines and animal models.

### 7.1 In vitro studies using cell lines

Table 2 shows the recent preclinical anticancer studies of 6‐shogaol.

The five most lethal cancers are breast, lung, colon, liver, and gastric. Current therapies can be effective against cervical cancer (CC) if diagnosed early; however, 341,831 deaths due to this cancer were recorded in 2020, which justifies the study of alternative therapies (Sung et al., 2021). In this context, HeLa and SiHa cells were used to determine the anticancer effect of 6‐shogaol. The results suggested that this compound inhibits proliferation and migration, induces apoptosis, and arrests the cell cycle at the G2/M phase of CC cells. At a molecular level, 6‐shogaol downregulates the expression of p‐PI3K, p‐Akt, and p‐mTOR. This phenolic compound might activate autophagy at a cellular level because it increases the number of autophagic vesicles and upregulates the microtubule‐associated protein light chain 3 (LC3) and Beclin1 levels. In contrast, it downregulates the expression of p62 (Pei et al., 2021a).

A network pharmacology approach identified that HSP90AA1, HRAS, EGFR, SRC, CASP‐3, HSP90AA1, MTOR, MAPK‐1, MDM2, and ESR1 might be target genes of 6‐shogaol, resulting in apoptosis induction and inhibition of cellular proliferation, growth, and migration, which might be associated with overall survival of CC patients (Elasbali et al., 2023). However, these bioinformatic results must be confirmed in cellular and animal models.

Among the gynecological cancers, ovarian cancer is the fifth‐most common. The effect of 6‐shogaol was tested in A2780, OVCAR‐3, Caov‐3, and SK‐OV‐3 human ovarian cancer cell lines. In A2780 and OVCAR‐3 cells, this compound triggered endoplasmic reticulum stress and apoptosis through upregulation of Nox4 expression, ROS generation, and Ca^2+^ release. This treatment also decreased cell viability and caspase‐3 activity. Moreover, it upregulates the expression of GRP78, p‐PERK, p‐eIF2α, ATF‐4, CHOP, and DR5. Interestingly, 6‐shogaol induces apoptosis in gefitinib‐resistant models by inhibiting the epithelial‐to‐mesenchymal transition (EMT) phenotype (Kim & Lee, 2023). In the A2780 ovarian cancer cell line, 6‐shogaol induced cytotoxicity, apoptosis, and ROS generation. Additionally, this phytochemical compound inhibited the translocation of the signal transducer and activator of transcription 3 (STAT3), resulting in the downregulation of the expression of PCNA, cyclin‐D1, B‐cell lymphoma 2 (Bcl‐2), Bax, caspase‐3, and caspase‐9 (Liang et al., 2019).

Endometrial cancer is one of the most common diseases associated with gynecological conditions. 6‐Shogaol inhibits the proliferation of human endometrial carcinoma Ishikawa cells by arresting the cell cycle at the G2/M phase. Additionally, this compound triggers apoptosis and ROS production. At molecular levels, it activates ER response biomarkers (Ma et al., 2020).

With 2,261,419 new cases yearly, breast cancer is women's most frequently diagnosed (Sung et al., 2021). In this context, 6‐shogaol has been shown to kill breast cancer monolayer cells (MCF‐7) and cancer‐stem cell‐like spheroid culture. Likewise, 6‐shogaol arrests the cell cycle in the G2/M phase in these cells. Interestingly, the doses of 6‐shogaol that caused this effect were not toxic to noncancerous cells (Ray et al., 2015). In the MDA‐MB‐231 cell line, this phytochemical compound inhibits cell invasion by reducing the nuclear factor κB (NF‐κB) transcriptional activity, resulting in a decreased activation of metalloproteinase‐9 (MMP‐9) gene transcription (Ling et al., 2010). Moreover, 6‐shogaol prevents cancer progression by inhibiting the secretion of CC‐chemokine ligand 2 (CCL2) in tumor‐associated dendritic cells (TADCs) (Hsu et al., 2015). The same results were observed in the lung cancer cell line (A549) (Hsu et al., 2015). Lung cancer deaths were estimated at 1,796,144 worldwide in 2020 (Sung et al., 2021). The evidence suggested that the antiproliferative effect of 6‐shogaol depends on cellular type in NSCLC cell lines. Interestingly, this compound arrests the cell cycle at different phases depending on the cell line. For example, it causes arrest at the G1 phase in NCI‐H1650 and in the G2/M phase in NCI‐H520 cells (Kim et al., 2014). Moreover, in human NSCLC 459 cells (an NSCLC cell line), 6‐shogaol induces autophagic cell death by inhibiting AKT/mTOR signaling pathway (Hung et al., 2009). In addition, in A549 cells, it induces apoptosis and arrests the cellular cycle at G0/G1 phase through reducing levels of Bcl‐2 and survivin, while increasing the expression of Bax and disrupting the function of the mitochondria. Therefore, 6‐shogaol cytotoxicity over A549 cells is due to the cell cycle arrest and mitochondria‐mediated apoptosis (Mulati et al., 2022).

Prostate cancer has a high incidence among men, with 1,414,259 new cases worldwide in 2020 (Sung et al., 2021). MTT assay and flow cytometry showed that 6‐shogaol (10, 20, and 40 μM) significantly inhibits the growth and induces apoptosis of human prostate cancer cell lines (LNCaP, DU145, and PC3). Similar results were observed in a prostate cancer cell line from mice (HMVP2) (Saha et al., 2014). Interestingly, this phytochemical compound reduced the survival of both androgen‐dependent (LNCaP) and androgen‐independent (DU145 and PC‐3) cell lines (Saha et al., 2014), suggesting that this male hormone did not affect the anticancer properties of 6‐shogaol.

Head and neck squamous carcinomas might be treatable if diagnosed early. Among this type of cancer, squamous cell carcinoma of the larynx is the most frequent. Concerning this, 6‐shogaol (5–50 μg) inhibited the proliferation of the Hep‐2 cell line by inducing oxidative stress‐mediated mitochondria‐dependent apoptosis (Annamalai et al., 2016). In vitro studies using squamous cell carcinoma cell lines of the tongue (SCC25 and CAL27) indicate that 6‐shogaol reduces proliferation and induces apoptosis dose dependently, possibly through downregulation of survivin. Moreover, this compound enhances radiosensitivity in carcinoma cell lines (Kotowski et al., 2018).

In human colorectal cancer cell lines (HCT‐116 and SW‐480), 6‐shogaol inhibited cell growth and arrested the cell cycle at the G2/M phase. Additionally, this phenolic compound induces higher apoptotic cell death and cell cycle arrest in p53+/+ cells than in p53−/− cells. Since 6‐shogaol upregulates the expression of p53, p21waf1/cip1, and GADD45α while downregulating the expression of mitotic cyclin‐dependent kinase (cdc2) and M‐phase‐inducer phosphatase 1 (cdc25A), it is possible that p21waf1/cip1 and GADD45α were activated by this bioactive compound in the p53‐dependent pathway (Qi et al., 2015). In the primary colorectal tumor cell line (SW480), 6‐shogaol induces higher cell death through the autophagy‐ and apoptosis‐related pathways than in a metastatic cell line (SW620). Furthermore, the chemotherapeutic agents such as 5‐fluorouracil, irinotecan, and oxaliplatin in combination with 6‐shogaol enhanced their cytotoxic effect by increasing the apoptosis and autophagy in hypoxic/glycemic conditions (Woźniak et al., 2020).

Moreover, M2 and M3 (cysteine‐conjugated metabolites) are 6‐shogaol analogs that produced a more substantial apoptotic effect in human colon cancer cell lines (HCT‐116) and human lung cancer cell lines (H‐1299) and no toxicity toward noncancer cells (human colon and lung fibroblast cells) when compared with 6‐shogaol. Interestingly, these 6‐shogaol analogs' stereoisomers are irrelevant to apoptotic cell death (Zhu et al., 2013).

Different administration forms, such as micelles carrying 6‐shogaol, have been evaluated regarding their anticancer properties. According to an oral pharmacokinetic study, micelles loaded with 6‐shogaol enhanced the delivery efficiency, oral bioavailability, and distribution in the liver regarding oral administration of 6‐shogaol. Remarkably, these micelles were endocytosed by a human hepatoma cell line (HepG2), causing a stronger inhibition of cell proliferation concerning free 6‐shogaol (Zhang et al., 2019). In liver cancer cells resistant to tumor necrosis factor (TNF)‐related apoptosis‐inducing ligand (TRAIL) (Huh7), 6‐shogaol induced attenuation of autophagy flux sensitizing to apoptosis via p53 and ROS in combination with TRAIL. The combined treatment of 6‐shogaol and TRAIL attenuated cell viability, increased cell death, upregulated the expression of p53, and increased the breakdown of caspase‐3 and caspase‐8, compared to single treatments. 6‐Shogaol upregulates p62 and the microtubule‐associated proteins 1A/1B light‐chain 3B‐II levels (Nazim & Park, 2019).

In human keratinocyte cell lines (HaCaT) and male C57BL/6 mice, 6‐shogaol reduced the production of IL‐1β, IL‐6, IL‐8, and TNF induced by ultraviolet B irradiation. The release of cytokines and chemokines function as activators of neutrophils, basophils, monocytes, and macrophages. Therefore, inhibiting inflammatory mediators in vivo and in vitro through 6‐shogaol treatment results in anti‐inflammatory activity in irradiated HaCaT (Guahk et al., 2010).

Finally, most consulted references agreed that 6‐shogaol has the highest potency to inhibit cancer cell growth compared with 6‐gingerol and 6‐paradol, or other ginger natural compounds.

### 7.2 In vivo studies in animal models

In mice models, 6‐shogaol is metabolized by the mercapturic acid pathway, and about 90% of the consumed compound was absorbed in the gastrointestinal and excreted more in feces than in urine (Table 2; Asami et al., 2010; Chen et al., 2012). In this model, cysteine‐conjugated shogaols were most toxic to cancer cells than the normal colon fibroblasts cells and caused cell death via activation of the mitochondrial apoptotic pathway (Fu et al., 2014).

In another study, female BALB/c nude mice were injected with HeLa cells (1 × 10^7^ cells/mL) to produce a CC model. Subsequently, these mice were treated daily with 6‐shogaol (12.5 and 50 mg/kg) for 21 days. Evidence suggested that this phenolic compound prevented tumor growth and triggered apoptosis, producing minimal secondary effects on weight loss and organ damage (Pei et al., 2021a).

Female athymic BALB/c nude mice (nu/nu) were used as ovarian cancer models by inoculation with A2780 human ovarian cancer cell line (1 × 10^7^ cells) and then treated with 6‐shogaol (40 or 60 mg/kg) for 2 days (Kim & Lee, 2023). The findings indicated a decrease in the tumor sizes with 6‐shogaol at least sixfold lower than controls. However, the authors did not show an analysis at the molecular level that complemented their findings using cancer cell lines.

Also, BALB/c female nude mice were injected with Ishikawa cells (endometrial cancer cell line) and treated with 6‐shogaol, inhibiting tumor growth without affecting the body weights compared with controls. Immunohistochemical evidence showed that the biomarkers of cell proliferation were significantly decreased after 6‐shogaol administration without toxicity (Ma et al., 2020).

In the hamster model with oral squamous cell carcinoma, 6‐shogaol enhanced the activity of cytochrome p450 (Cyt‐p450) and cytochrome b5 (Cyt‐b5). In addition, this phytochemical compound induced the overexpression of Bcl‐2 and decreased the amount of Bax, inhibiting lipid peroxidation and improving antioxidant status (Kathiresan & Govindhan, 2016).

Moreover, 6‐shogaol decreased metastasis in BALB/c mice models of lung and breast cancer (Hsu et al., 2015). BALB/c mice models implanted with 4 T1 mammary tumor cells were treated with 6‐shogaol (30 mg/kg of body weight) for 14–21 days, decreasing CCL2 in TADCs and reducing metastasis. Similar findings were observed in the C57BL mice model implanted with Lewis lung carcinoma cells. The authors suggested that the inhibition of metastasis in breast and lung in vivo models was associated with the inhibition of STAT3 in TADCs (Hsu et al., 2015). Moreover, 6‐shogaol has a proapoptotic activity and reduces the growth of NSCLC in a BALB/c xenograft mice model subcutaneously injected with NCI‐H1650 cells. Evidence indicates that the phosphorylation of STAT3 was reduced, leading to a downregulation of the expression of gene products associated with this transcriptional factor, such as cyclin D1, cyclin D3, and c‐Myc (Kim et al., 2014). Moreover, 6‐shogaol inhibits the growth NSCLC cells both in vitro and in vivo. A high concentration of 6‐shogaol induces toxicity in zebrafish embryos, revealed by increased mortality and hatching inhibition. However, no toxicity effects were observed in low concentrations, suggesting this phenolic compound's anticancer potential (Mulati et al., 2022).

Xenograft models of human colon cancer cells (HCT‐116‐Luc) treated with 6‐shogaol (15 mg/kg) showed a significant reduction in tumor growth. At a cellular level, this compound arrests the cell cycle at the G2/M phase and induces apoptosis in a dose‐dependent manner. These phenomena were accompanied by the upregulation of the p53‐dependent pathway, decreasing the expression of cdc2 and cdc25A while p21^waf1/cip1^ increases its amount (Qi et al., 2015).

Furthermore, Saha et al. (Elasbali et al., 2023) revealed that 6‐shogaol inhibits tumor growth in an in vivo prostate cancer model. In their experiment, FVB/N male mice were subcutaneously injected with HMVP2 cells (prostate cancer cells), then animals were intraperitoneally treated with 6‐shogaol (50 and 100 mg/kg body weight) for 32 days. According to their findings, 6‐shogaol inhibited the growth of HMVP2 cells and had a proapoptotic effect. The molecular mechanism of the apoptotic effect of this compound is associated with inhibiting the constitutive or IL‐6‐induced phosphorylation of STAT3 that resulted in the downregulation of the levels of pSTAT3, cyclin D1, and survivin (Saha et al., 2014).

As mentioned above, the antitumor activities of 6‐shogaol are limited due to its poor water solubility. Thus, research focuses on developing novel administration methods using nanotechnology. In this way, PEG micelles and a linoleic acid derivative were used to enhance the bioavailability and solubility of this phenolic compound in male Sprague–Dawley rats. Interestingly, micelles loaded with 6‐shogaol increased bioavailability by 3.2‐fold, augmented tissue distribution in the liver and brain, and enhanced hepatoprotective action compared with free 6‐shogaol treatment (Zhang et al., 2019). Moreover, 6‐shogaol induced hepatoprotective activity in a hepatotoxic Kunming mice model when it was loaded into micelles for administration. At molecular levels, these micelles induced higher activities of glutathione peroxidase and superoxide dismutase while downregulating the levels of malondialdehyde (MDA) in the liver of mice, compared with the free 6‐shogaol administration. Encapsulated 6‐shogaol reduced the activities of AST and ALT in serum, decreased MDA levels in the liver, and upregulated the activities of GSH‐Px and T‐SOD, suggesting that 6‐shogaol micelles protect the liver (Zhang et al., 2019). Evidence suggested that the principal participation of 6‐shogaol in vitro systems is related to the STAT3, NF‐κB, ATK, and mTOR signaling pathways, and in vivo systems, the oxidative stress pathways might also be involved in the antitumor effect of this compound.

## 8 MECHANISM OF ANTITUMOR ACTION OF 6‐SHOGAOL

**FIGURE 2:**
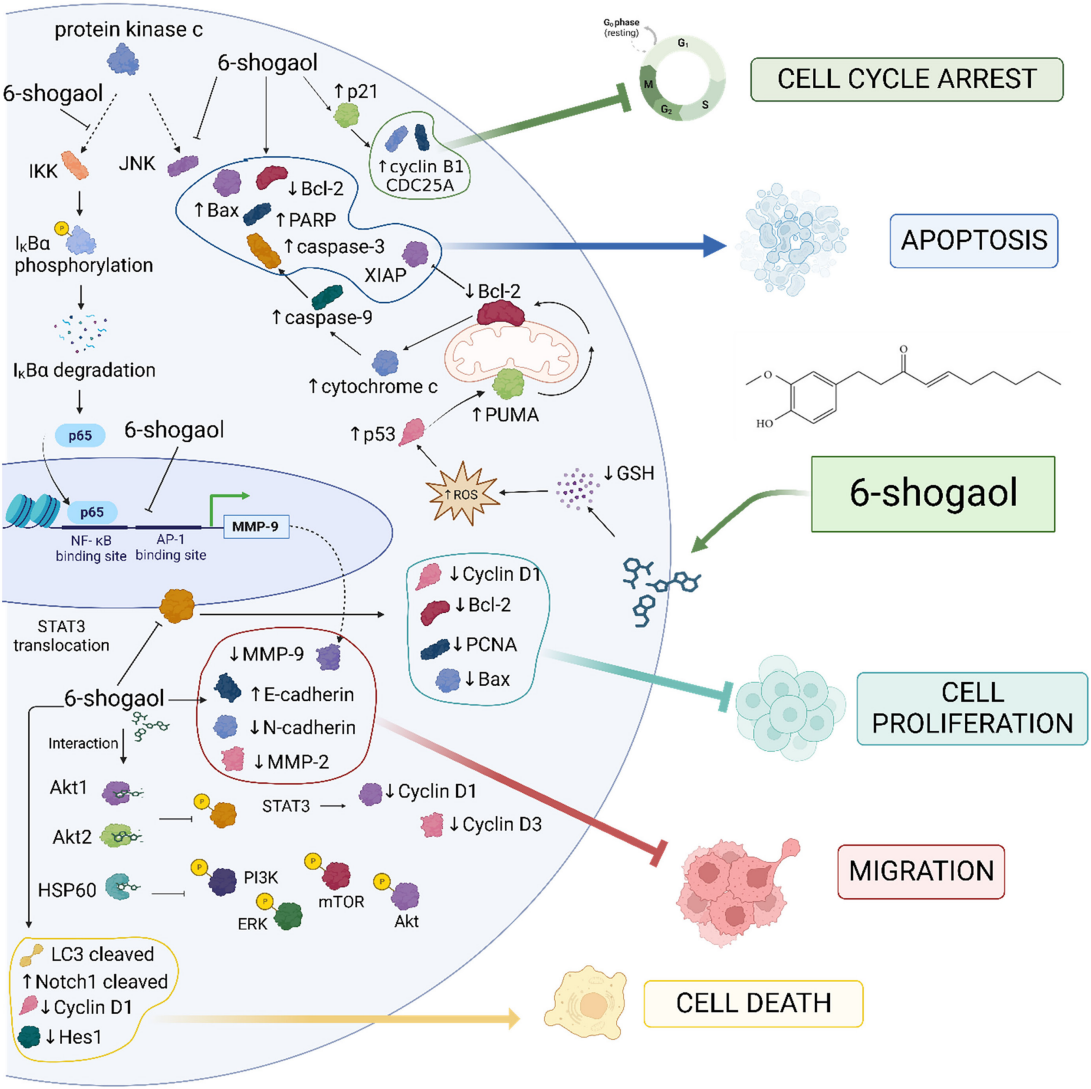
Molecular mechanism of antitumoral properties of 6‐shogaol. The anticancer activities of DIM (a) and I3C (b) involve the positive (↑) and/or negative (↓) regulation of protein expression and activities, inducing (→) or repressing (┤) cellular processes considered characteristics of cancer. Akt1, protein kinase B or PKB 1; Akt2, protein kinase B or PKB 2; ERK, extracellular signal‐regulated kinases; Hes1, hairy and enhancer of split‐1; HSP60, heat shock protein; IKK, inhibitor of nuclear factor kappa‐B kinase; IκBα, inhibitor of nuclear factor kappa‐B alpha; JNK, c‐Jun N‐terminal kinase; LC3, microtubule‐associated protein light chain 3; MMP‐2, metalloproteinase‐2; MMP‐9, metalloproteinase‐9; NF‐κB, nuclear factor kappa‐B; Notch, Notch signaling pathway; PI3K, phosphoinositide 3‐kinase; PUMA, p53 upregulated modulator of apoptosis; STAT3, signal transducer and activator of transcription 3; XIAP, X‐linked inhibitor of apoptosis protein. Created with BioRender.com (Accessed 5 May 2023).

6‐Shogaol enters the cell by passive diffusion (Almatroudi et al., 2019). The molecular mechanism of 6‐shogaol associated with anticancer activities is summarized in Figure 2.

In CC, 6‐shogaol triggers apoptosis independent of autophagy through the ROS‐mediated PI3K/Akt/mTOR signaling pathway. Interestingly, this phenolic compound arrests the cell cycle by a mechanism associated with the upregulation of p21, a regulator molecule of the G2/M phase that binds to CDKs‐cyclins. Then, p21 downregulates the expression of cyclin B1 and CDC25A. Furthermore, 6‐shogaol reduces the mitochondrial membrane potential resulting in the upregulation of mitochondrial apoptotic proteins (Bax, PARP, and cytochrome c), the downregulation of antiapoptotic protein (Bcl‐2), and finally, promoting the apoptosis through the mitochondria pathway. Moreover, 6‐shogaol increases intracellular ROS levels, suggesting that apoptosis induction is also a ROS‐dependent mechanism in HeLa and SiHa cells. Interestingly, this phenolic compound upregulates E‐cadherin's expression while downregulating N‐cadherin's expression, inhibiting cellular migration. In addition, 6‐shogaol decreases the levels of MMP‐2 and MMP‐9, which might be involved in migration reduction (Pei et al., 2021a).

In ovarian cancer, 6‐shogaol increases the concentration of ER stress markers such as GRP78, p‐PERK, p‐eIF2α, ATF4, and CHOP. Subsequently, activated CHOP binds to the DR5 promoter, regulating its expression and promoting caspase‐3‐dependent apoptotic cell death. Therefore, 6‐shogaol provokes cell death by upregulation of Nox4 and release of ROS, which subsequently stimulates ER stress via intracellular Ca^2+^ release. In addition, 6‐shogaol in combination with gefitinib decreases the resistance of the drug by ER stress activation, inhibition of the expression of E‐cadherin, and increase in the expression of N‐cadherin, vimentin, Slug, and Snail, which results in the suppression of EMT cellular event (Kim & Lee, 2023). At the cellular level, this phenolic compound promotes morphological changes such as membrane blubbing and nuclear fragmentation associated with the apoptotic process. In addition, 6‐shogaol increases the expression of Bax, caspase‐3, and caspase‐9 in ovarian cancer cell lines but decreases Bcl‐2 levels. These findings suggested that 6‐shogaol provokes cytotoxicity and apoptosis, enhances ROS production, and prevents the translocation of STAT3, which reduces the overexpression of PCNA, cyclin‐D1, and Bcl‐2, and downregulates the concentration of Bax, caspase‐3, and caspase‐9 in ovarian cancer cell lines (A2780). Therefore, 6‐shogaol inhibits cell proliferation through the JAK/STAT3 signaling pathway (Liang et al., 2019).

Furthermore, 6‐shogaol decreases cell viability and stimulates cell cycle arrest in the G2/M phase in Ishikawa cells or Ishikawa cell xenograft model. In this context, this phenolic compound decreased the mRNA expression of cyclin E, CDK2, cyclin B, and cdc2 and increased the expression of p53 and p21. In Ishikawa cells, 6‐shogaol treatment increased apoptosis and decreased the mitochondrial membrane potential. Moreover, the levels of Fission 1 protein and the dynamin‐related protein 1 were upregulated, while the expression of mitofusin 1 and 2 and intermembrane proteins optic atrophy1 was decreased. Also, 6‐shogaol enhances ROS production, activating ER response biomarkers such as GRP78/BIP, ATF‐6, IRE1α, P‐JNK, CHOP, and caspase‐12, while the expression of IRE1α decreases (Ma et al., 2020).

On the other hand, 6‐shogaol downregulates GSH‐inducing ROS generation. This oxidative stress causes activation of the p53 pathway. 6‐Shogaol increased the rate of p53, which resulted in a p53‐upregulated modulator of apoptosis (PUMA) induction via p53 translocation to the mitochondrial surface, leading to a Bcl‐2 downregulation. This decrease in Bcl‐2 cause a release of cytochrome c and the inhibition of the interaction of X‐linked inhibitor of apoptosis protein (XIAP) with caspases, and finally, activation and cleave of caspase‐9 and ‐3, resulting in apoptosis of human colon cancer cells (Fu et al., 2014).

In colorectal cancer cells, 6‐shogaol increases the levels of p53, the CDK inhibitor p21^waf1/cip1^, and GADD45α and decreases cdc2 and cdc25A. Moreover, 6‐shogaol binds to the active domain of Bcl‐2, preventing its antiapoptotic function and resulting in apoptosis induction through the mitochondrial pathway (Qi et al., 2015). Therefore, 6‐shogaol arrest the G2/M cell cycle and induce apoptosis through modulation of the expression of p53/p21‐cdc2/cdc25A, resulting in the death of colorectal cancer cells.

In breast cancer monolayer and spheroid cell lines, 6‐shogaol causes cytoplasmic vacuole formation and cleaving of the LC3. Furthermore, 25 μM of 6‐shogaol induced the expression of cleaved Notch1 and reduced the expression of Hes1 and cyclin D1, disintegrating breast cancer spheroids and, finally, causing cell death (Ray et al., 2015).

In addition, in the MDA‐MB‐231 cell line, phorbol 12‐myristate 13‐acetate (PMA) induces expression of MMP‐9, resulting in cell invasion through NF‐κB and ERK‐dependent AP‐1 transcriptional activation pathways. In this way, 6‐shogaol reduces cell invasion through NF‐κB activation cascade by the inhibition of phosphorylation of IKK complex (IKKα and IKKβ subunits), blocking the IκBα phosphorylation and preventing its proteasomal degradation. All these events decrease p65 nuclear translocation and subsequently cause the inhibition of NF‐κB transcriptional activation, resulting in downregulation of the expression of MMP‐9 and, finally, reducing breast cancer cell invasion (Ling et al., 2010).

In lung and breast cancer cells, 6‐shogaol inhibits the phosphorylation of STAT3 and decreases the CCL2 expression, preventing the effects of TADCs on tumorigenesis and metastasis in vivo and in vitro. CCL2 promotes progression, migration, invasion, and immune‐suppressive immune cell infiltration in cancer cells; thus, tumorigenesis and progression decrease in the presence of this phytochemical. Furthermore, 6‐shogaol decreases the accumulation of immune‐suppressive cells (Hsu et al., 2015).

In NSCLC cells, 6‐shogaol interacted with Akt1 and Akt2, inhibiting their phosphorylation activity. The inhibition of Akt resulted in attenuated phosphorylation of STAT3, a transcriptional regulator of cyclin D1 and D3. However, this compound did not affect the activity of PI3‐K or mTOR in NCI‐H1650 cells. Likewise, 6‐shogaol reduces the anchorage‐independent growth of Akt‐overexpressing cells (Kim et al., 2014). Moreover, the network pharmacology approach suggested that ERK, STAT3, PI3K, Akt, and mTOR signaling pathways might be involved in the anticancer activity of 6‐shogaol on NSCLC cells. Experimental evidence indicates that 6‐shogaol decreased Bcl‐2 expression, activated the expression of Bax, and disrupted the MMPs, suggesting that it induces apoptosis through a mitochondrial pathway. Interestingly, the binding energy of 6‐shogaol to HSP60 (−22.39 ± 1.68 kcal/mol) suggested that the two molecules formed a strong complex that resulted in HSP60 degradation via the proteasome. In addition, the 6‐shogaol/HSP60 complex inhibited the phosphorylation of ERK, STAT3, PI3K, Akt, and mTOR, but this compound did not influence the protein levels in A549 cells. Moreover, the 6‐shogaol's anticancer activities on NSCLC cells were enhanced with Taxol (Mulati et al., 2022).

6‐Shogaol downregulated the expression of STAT3‐ and NF‐κB‐regulated target genes such as cyclin D in prostate cancer cells (Saha et al., 2014). In line with these results, 6‐shogaol diminished the constitutive phosphorylation of STAT3 in human prostate cancer cell lines (DU145 and HMVP2) and decreased the IL‐6‐induced activation of STAT3 in LNCaP (Saha et al., 2014). Interestingly, 6‐shogaol also inhibits the activation of JAK2 and Src, upstream regulation kinases of STAT3 activation (Johnston & Grandis, 2011; Saha et al., 2014). In addition, 6‐shogaol inhibits the constitutive NF‐κB activity and the TNF‐α‐induced phosphorylation of NF‐κB in prostate cancer cell lines. As a result of NF‐κB and STAT3 inhibition, the levels of cyclin D, cMyc, and survivin were reduced. Furthermore, 6‐shogaol inhibits NF‐κBp65 nuclear localization and decreases the TNF‐α‐induced phosphorylation of IκBα, an upstream regulator of NF‐κB (Saha et al., 2014). Therefore, the inhibition of NF‐κB is through the inhibitor of nuclear factor kappa‐B kinase (IKK)/IκB complex (Yemelyanov et al., 2006).

Regarding head and neck cancer, the evidence suggested that 6‐shogaol might be a promising anticancer agent. Regular consumption of tobacco and alcohol is related to the apparition of squamous cell carcinoma of the larynx. In this way, 6‐shogaol enhanced ROS production, which altered mitochondrial membrane potential, increasing oxidative DNA damage and nuclear fragmentation. Moreover, this phytochemical decreased Bcl‐2, increased Bax, and released cytochrome c from mitochondria, activating caspase‐3 and ‐9 (Annamalai et al., 2016). An increase in apoptosis through a downregulation of survivin was reported for the tongue's squamous cell carcinoma cell lines treated with 6‐shogaol. In contrast, 6‐shogaol did not affect Mcl‐1 expression, contributing to the apoptosis induction in this type of head and neck cancer cell lines (Kotowski et al., 2018). All this evidence suggested that 6‐shogaol provokes apoptosis in a dose‐dependent and cell‐type‐specific way.

In oral squamous cell carcinoma, 6‐shogaol induces upregulation of COX‐2, iNOS, TNF‐α, IL‐1, and IL‐6 and increases the amount of cell proliferative biomarkers such as cyclin D, PCNA, and Ki‐67. Furthermore, this compound inactivated the phosphorylation of IκB, c‐jun, and c‐fos, inhibiting the transcriptional activation of NF‐κB and AP‐1, which inhibited inflammation and cell proliferation (Annamalai & Suresh, 2018). Furthermore, 6‐shogaol induces the aberrant activation of NF‐κB, AP‐1, IKKβ, c‐jun, and c‐fos (Annamalai & Suresh, 2018). 6‐Shogaol triggered apoptosis by inhibiting the EMT and EGFR/PI3K/Akt pathways in oral squamous cell carcinoma (Huang et al., 2021).

Compared with a single treatment, a combinatory regimen of 6‐shogaol with TRAIL upregulated cell death in human liver cancer cells (Huh7 and Hep3B). Moreover, this treatment increases the cleaved caspase‐3 and cleaved caspase‐8, indicating that combining those compounds increases autophagy compared to the single application. In addition, 6‐shogaol and TRAIL applied together increased p53 and autophagy‐associated proteins such as LC3‐II and p62, inhibiting autophagy flux. Therefore, this combinatory treatment induces apoptosis via p53 and ROS (Nazim & Park, 2019).

Finally, we cannot discard that the anticancer activity of 6‐shogaol could be attributed to unsaturated ketone moieties (Yang et al., 2018) that might give this phytochemical its high antioxidant capacity, modulating the activity of several signaling molecules. For example, it modulates the expression of NF‐κB, STAT3, MAPK, PI3K, ERK1/2, Akt, TNF‐α, COX‐2, cyclin D1, cdk, MMP‐9, survivin, cIAP‐1, XIAP, Bcl‐2, and caspases (Prasad & Tyagi, 2015). However, 6‐shogaol also inhibits the expression of PPARγ and C/EBPα, which regulate the adipogenesis and lipogenesis in mature 3 T3‐L1 adipocytes (Suk et al., 2016).

## 9 HUMAN CLINICAL STUDIES

The study investigated the clinical trials that reported determining the effects of 6‐shogaol in human. For this purpose, we reviewed the International Clinical Trials Registry Platform of the World Health Organization (https://trialsearch.who.int/). We found one clinical trial using the word “shogaol” registered on 8 December 2021 (https://trialsearch.who.int/Trial2.aspx?TrialID=IRCT20191221045837N4) (accessed on May 9, 2023). The Mashhad University of Medical Sciences was the primary sponsor of this clinical study, and its objective was the evaluation of the efficacy of ginger supplementation with pellet formulation in children with juvenile idiopathic arthritis. The intervention group recruited in the Islamic Republic of Iran received ginger pellets (containing ginger extract equivalent to 250 mg of ginger rhizome powder, standardized based on shogaol and phenolic acid contents) twice daily for 3 months. The analysis of groups included the severity of the illness assessed by a physician, the general well‐being of the patient, and functional ability, among others. Regrettably, no results have been posted so far.

We also searched human clinical trials in the ClinicalTrials.gov database (https://clinicaltrials.gov/), which includes over 451,538 research studies in 221 countries. Using the word “6‐shogaol,” we found one completed clinical trial (accessed on 9 May 2023). This study from France reported the pharmacokinetics assessing the bioavailability of gingerols and shogaols using five different ginger extracts, which started on 15 October 2018, and was completed on 7 December 2018. The sponsors of this trial were Naturex SA and Biofortis Mérieux NutriSciences, Saint‐Herblain, France. The clinical trial included the measure of the total gingeroids plasmic concentration before ginger extract administration and 8 and 24 h after administration in 20 adults from 18 to 55 years old. Furthermore, each analyte of ginger plasmatic concentration and its half‐time was identified. In addition, the half‐life of total gingeroids plasmatic concentrations was investigated. Although this clinical trial is marked as completed, no published results exist.

Finally, in 2008, human volunteers were enrolled in a clinical trial to determine the pharmacokinetics and tolerability of 6‐shogaol, 6‐, 8‐, and 10‐gingerol, and their conjugate metabolites. Volunteers received 100 mg to 2.0 g of ginger. After 15 min and 72 h, blood samples were taken to detect free analytes. Results indicate that after oral administration of ginger root, 6‐, 8‐, and 10‐gingerol, and 6‐shogaol were quickly absorbed and detected in serum as glucuronide and sulfate conjugate (Zick et al., 2008).

## 10 TOXICITY, SIDE EFFECTS, AND SAFETY

6‐Shogaol is an aromatic compound from ginger, widely used as a spice and herbal medicine. Although dietary compounds are attractive options for treating and preventing human diseases because human bodies have been accepting them for a long time, the possible toxicity and safety of 6‐shogaol to normal cells and organs have not been thoroughly evaluated, especially in human studies.

It has been described that 6‐shogaol has low toxicity in different types of normal cells and experimental animals. For example, 6‐shogaol had minimal effects on the viability of normal human colon (CCD‐18Co) and human lung (IMR‐90) cells at 5–20 μM concentrations, with IC_50_ values of 43.91 and 36.65, respectively (Kocarnik et al., 2022; Sung et al., 2021). Interestingly, several metabolites of 6‐shogaol had some anticancer activity and appeared less toxic than 6‐shogaol (Sung et al., 2021). Likewise, 6‐shogaol has shown a robust proapoptotic action in hepatocellular carcinoma cells (SMMC‐7721, BEL‐7404, and HepG2) with low toxicity in normal human liver cells (HL‐7702) at concentrations of 20, 40, and 80 μM (Siegel et al., 2022). Remarkably, experiments in mice xenografted with SMMC‐7721 cells confirmed the anticancer effects of 6‐shogaol. Moreover, no significant difference in body weights was found between groups treated with vehicle and 6‐shogaol, suggesting no substantial toxicity in vivo. Another study revealed that 6‐shogaol killed both breast cancer monolayer cells and spheroids at doses that did not harm noncancerous cells (HEK 293 and HaCaT cells), indicating selective toxicity against cancer cells. On the other hand, Han et al. investigated how 6‐shogaol affects human dermal fibroblasts (HDFs) exposed to hydrogen peroxide, which causes oxidative stress. They found that 6‐shogaol exhibited no toxicity in this cell line at 5–20 μM (Kumari & Dang, 2021). This result suggests that the compound is safe for topical use, corroborated by another similar study (National Cancer Institute, 2023). Likewise, a recent study evaluated the effects of administering 6‐shogaol in female BALB/c nude mice as a model (Basak et al., 2021). The authors observed no differences in body weight or histology of heart, liver, spleen, lung, and kidney between the group receiving 6‐shogaol and the group not, indicating no toxicity.

To our knowledge, no clinical trials have been reported on ClinicalTrials.gov exploring the safety or toxicity of 6‐shogaol in humans. Numerous clinical studies have evaluated the safety of consuming ginger or ginger extracts at different doses but not the isolated compound (Ali et al., 2008; da Silveira Vasconcelos et al., 2019; Dallavalle et al., 2020; Holohan et al., 2013; Nedungadi et al., 2018). Therefore, the safety of 6‐shogaol for human use is not well established. In this regard, some studies on its pharmacokinetics and metabolism in human subjects (Ballester et al., 2022; Pan et al., 2008; Zhang et al., 2021). Those studies demonstrated that 6‐shogaol is extensively metabolized in the body, and its metabolites may have different biological activities and toxicity profiles. For example, Qiu et al. (Ray et al., 2015) studied the molecular interactions of 6‐shogaol and other ginger components with human cytochrome P450 (CYP) 1A2, 2C9, 2C19, 2D6, and 3A4. They used computational methods and literature searches to estimate those ginger components' absorption, distribution, metabolism, excretion, and toxicity. Their study indicates that ginger components may affect the function and expression of various human CYPs, leading to drug elimination and effect changes.

Finally, since many studies have shown minimal toxicity or no side effects in normal cells and experimental animals, we could speculate that 6‐shogaol possesses a good safety profile at low and moderate concentrations that produce therapeutic results in distinct cancer models. However, clinical trials are needed to confirm 6‐shogaol's safety for human use, even though preclinical studies provide some clues.

## 11 CONCLUSION AND PERSPECTIVES

6‐Shogaol is an active component of ginger that has shown encouraging antitumor effects in various types of cancer cells, both in vitro and in vivo. 6‐Shogaol can inhibit cell proliferation and migration, induce cell cycle arrest and apoptosis, and modulate key signaling pathways involved in cancer development and progression, such as STAT3, NF‐κB, and PI3K/Akt/mTOR. Moreover, 6‐shogaol can prevent cancer development and lung metastasis by inhibiting the secretion of CCL2 in tumor‐associated dendritic cells, as well as enhance the anticancer effect of current chemotherapeutic agents such as 5‐fluorouracil, oxaliplatin, taxol, and irinotecan. In this regard, we propose some future directions for research on 6‐shogaol:

- To evaluate the antiangiogenic, proapoptotic, and antimetastatic effects of 6‐shogaol in different cancer cells and animal models and to identify the key molecular targets and regulators involved in these processes.
- Examine the binding mechanism and interaction sites of 6‐shogaol with HSP60 in NSCLC cells and explore its role in modulating the cellular stress response and mitochondrial function.
- To optimize the dose and administration route of 6‐shogaol for achieving the maximum antitumor effect in cervical carcinoma via PI3K/Akt/mTOR pathway and to examine its influence on the tumor microenvironment and immune system.
- To explore the effects of 6‐shogaol on the epigenetic regulation of gene expression and DNA methylation in cervical carcinoma cells and to examine its influence on the tumor suppressor genes and oncogenes.

Collectively, evidence suggests that 6‐shogaol possesses the properties of a chemopreventive and therapeutic agent that could be used as a complementary or alternative therapy for cancer treatment. However, additional investigations are needed to establish the molecular mechanisms of action of 6‐shogaol in different cancer types and stages and its pharmacokinetics, bioavailability, and safety in humans. Moreover, the synergistic or additive effects of 6‐shogaol with conventional chemotherapeutic agents or other natural compounds should be explored to enhance its efficacy and reduce toxicity. Developing novel formulations or delivery systems of 6‐shogaol could also improve its stability and bioactivity in vivo. Therefore, more research is warranted to fully exploit the potential of 6‐shogaol as a novel anticancer agent derived from ginger.

## AUTHOR CONTRIBUTIONS

**Gabriela Figueroa‐González:** Data curation (equal); investigation (equal); methodology (equal); writing – original draft (equal); writing – review and editing (equal). **Laura Itzel Quintas‐Granados:** Data curation (equal); investigation (equal); methodology (equal); writing – original draft (equal); writing – review and editing (equal). **Octavio Daniel Reyes‐Hernández:** Data curation (equal); investigation (equal); methodology (equal); writing – original draft (equal); writing – review and editing (equal). **Isaac H. Caballero‐Florán:** Data curation (equal); investigation (equal); methodology (equal); writing – original draft (equal); writing – review and editing (equal). **Sheila I. Peña‐Corona:** Data curation (equal); investigation (equal); methodology (equal); writing – original draft (equal); writing – review and editing (equal). **Hernán Cortés:** Data curation (equal); investigation (equal); methodology (equal); writing – original draft (equal); writing – review and editing (equal). **Gerardo Leyva‐Gómez:** Data curation (equal); investigation (equal); methodology (equal); supervision (equal); validation (equal); visualization (equal); writing – original draft (equal); writing – review and editing (equal). **Solomon Habtemariam:** Investigation (equal); methodology (equal); validation (equal); visualization (equal); writing – original draft (equal); writing – review and editing (equal). **Javad Sharifi‐Rad:** Conceptualization (equal); data curation (equal); investigation (equal); methodology (equal); project administration (equal); supervision (equal); validation (equal); visualization (equal); writing – original draft (equal); writing – review and editing (equal).

## CONFLICT OF INTEREST STATEMENT

The authors have no relevant financial or other conflicts of interest.

## Data Availability

Data sharing is not applicable to this article as no new data were created or analyzed in this study.
